# Distribution of coniferin in freeze-fixed stem of *Ginkgo biloba L.* by cryo-TOF-SIMS/SEM

**DOI:** 10.1038/srep31525

**Published:** 2016-08-11

**Authors:** Dan Aoki, Yuto Hanaya, Takuya Akita, Yasuyuki Matsushita, Masato Yoshida, Katsushi Kuroda, Sachie Yagami, Ruka Takama, Kazuhiko Fukushima

**Affiliations:** 1 Graduate School of Bioagricultural Sciences, Nagoya University, Furo-cho, Chikusa-ku, Nagoya, Aichi 464-8601, Japan; 2 Department of Wood Properties and Processing, Forestry and Forest Products Research Institute, 1 Matsunosato, Tsukuba, Ibaraki 305-8687, Japan

## Abstract

To clarify the role of coniferin *in planta*, semi-quantitative cellular distribution of coniferin in quick-frozen *Ginkgo biloba L*. (ginkgo) was visualized by cryo time-of-flight secondary ion mass spectrometry and scanning electron microscopy (cryo-TOF-SIMS/SEM) analysis. The amount and rough distribution of coniferin were confirmed through quantitative chromatography measurement using serial tangential sections of the freeze-fixed ginkgo stem. The lignification stage of the sample was estimated using microscopic observations. Coniferin distribution visualized at the transverse and radial surfaces of freeze-fixed ginkgo stem suggested that coniferin is stored in the vacuoles, and showed good agreement with the assimilation timing of coniferin to lignin in differentiating xylem. Consequently, it is suggested that coniferin is stored in the tracheid cells of differentiating xylem and is a lignin precursor.

Lignification is an important differentiation process in plant cell walls in which lignin is deposited in the polysaccharide matrix^1^,^2^. The whole structure of lignin is still unclear, and the controlling mechanism of lignin biosynthesis is also controversial^3^. Monolignol is a monomer unit of lignin, and its glucoside is a promising candidate for the lignin precursor^1^,^4^,^5^,^6^,^7^,^8^. Previous studies have showed that administered monolignol glucoside is assimilated into lignin without any alteration to the natural lignification process^9^,^10^,^11^,^12^,^13^,^14^,^15^,^16^,^17^. Furthermore, the endogenous monolignol glucoside is detected in natural plants in their growth period^6^,^18^,^19^,^20^,^21^,^22^. However, the discussion related to monolignol glucoside, in other words, the point that monolignol glucoside is the real precursor of lignin or not is ongoing^3^ because its individual cellular distribution *in planta* is still unclear.

Many studies have attempted to visualize the distribution of monolignol glucoside. Two recent studies conducted by using Raman microscopy^23^ and matrix-assisted laser desorption/ionization mass spectrometric imaging (MALDI-MSI)^24^ suggested the coniferin distribution in the lumina, cell corner middle lamella, and secondary wall of differentiating tracheid cells; however, the lateral resolution of MALDI-MSI was not sufficient to assess the individual cellular distribution of target chemicals and their experimental procedures required drying and pre-treatment of the sample.

The difficulty of the visualization of monolignol glucosides is due largely to their water solubility. The positional information of water-soluble chemicals might be lost or altered as a result of pre-treatment processes, such as drying, histological fixation, dyeing, and resin-embedding process for microscopic observations.

To overcome this problem, in this study, freeze-fixed samples are measured by time-of-flight secondary ion mass spectrometry (TOF-SIMS) equipped with cooling stage. In TOF-SIMS measurements, the main polymer components and various extractives are detected as molecule and/or fragment ions and their distribution can be visualized with submicron lateral resolution^25^,^26^. TOF-SIMS analysis with a cooling sample stage, which is known as cryo-TOF-SIMS, allows us to analyse the frozen-hydrated sample and visualize the distribution of water-soluble chemicals in biological tissues. The cryo-TOF-SIMS technique has been developed over several decades^27^; nevertheless, only a small number of reports exist of the use of these methods for higher plants^28^,^29^,^30^,^31^,^32^,^33^,^34^,^35^ and for the analysis of organic biomolecules in the plant^35^.

In this study, cryo-TOF-SIMS analysis was applied to the transverse and radial surfaces of quick-frozen stem of *Ginkgo biloba L*. (ginkgo) to visualize the cellular distribution of water-soluble coniferin. Ginkgo is a gymnosperm, and the main monomer unit of its lignin formation is coniferyl alcohol which is the aglycon unit of coniferin. The characteristic secondary ion of coniferin was determined by a comparative study of standard chemicals having a similar molecular weight. The amount and rough distribution of coniferin were confirmed by the quantitative chromatography measurement using serial tangential sections of the freeze-fixed ginkgo stem. The lignification stage of the sample was estimated by microscopic observations.

The cellular distribution of coniferin revealed by cryo-TOF-SIMS analysis was consonant with the lignification stages of the tracheid cells. Furthermore, the semi-quantitative distribution of coniferin was investigated with the two-step assimilation timing of coniferin to lignin demonstrated by ^14^C-labelled coniferin administration experiments^11^. Consequently, it is suggested that coniferin was stored in the tracheid cells of differentiating xylem and used as a lignin precursor.

## Results and Discussion

### Cryo-TOF-SIMS standard spectra

To determine the characteristic secondary ion of coniferin, standard chemicals were measured by cryo-TOF-SIMS. In TOF-SIMS, secondary ions often arise differently in different chemical conditions, and this effect is known as a matrix effect^25^. Therefore, KCl was added to the aqueous solutions of standard chemicals to approximate their chemical condition to that *in planta*, as potassium is the most abundant inorganic cation in plants^36^,^37^, and inorganic metals can readily ionize and affect the neighbouring organic chemicals in TOF-SIMS measurements^25^,^26^.

**Figure 1:**
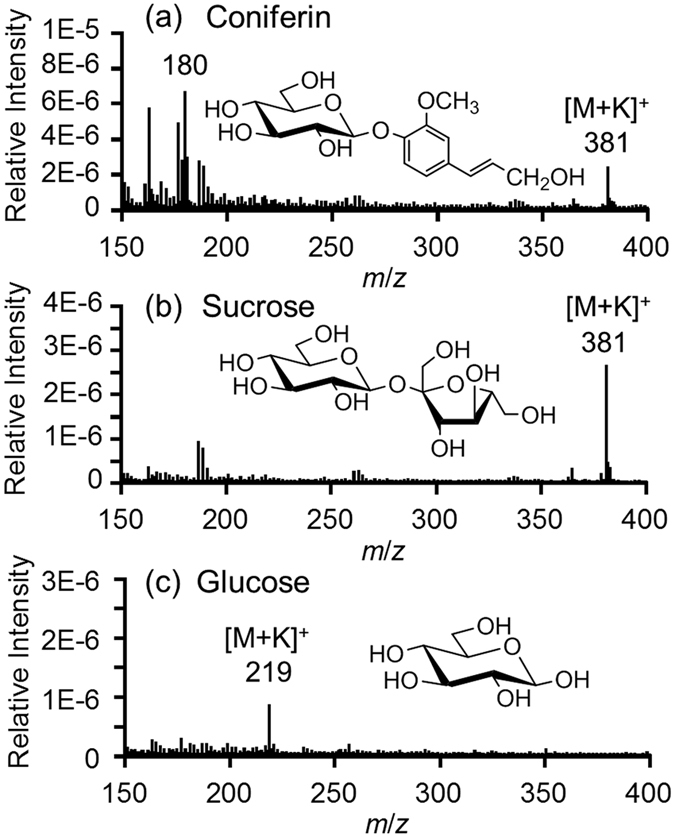
Cryo-TOF-SIMS spectra and chemical structures of (**a)**coniferin, (**b**) sucrose, and (**c**)glucose. All chemicals were dissolved at 1 mM concentration in 10 mM KCl aqueous solution and frozen for measurement.

In this study, glucose, fructose, sucrose, and coniferyl alcohol were selected as standard chemicals whose secondary ion peaks might overlap with that of coniferin. The cryo-TOF-SIMS spectrum of fructose was almost the same as that of glucose (Supplementary Fig. 1), and coniferyl alcohol was not detected in considerable amounts (Supplementary Fig. 2) by HPLC measurement. Resultant standard cryo-TOF-SIMS spectra of coniferin, sucrose, and glucose are shown in Fig. 1.

Major secondary ions in the coniferin spectrum were the potassium-adduct [M+K]^+^ ion of mass to charge ratio (*m*/*z*) 381 and the fragment ion of *m*/*z* 180 (Fig. 1a). To confirm the structure of the *m*/*z* 180 ion, coniferin labelled with ^13^C at the aglycon unit was synthesized and measured by cryo-TOF-SIMS (Supplementary Fig. 3). As a result, ions of *m*/*z* 382 and 181 were obtained instead of ions of *m*/*z* 381 and 180. Therefore, the *m*/*z* 180 ion was assigned to the fragment ion derived from the aglycon unit of coniferin.

Sucrose showed the highest secondary ion peak at *m*/*z* 381 as the [M+K]^+^ ion (Fig. 1b). Sucrose (C_12_H_22_O_11_, 342.12) has a very similar molecular mass to that of coniferin (C_16_H_22_O_8_, 342.13). It was difficult to distinguish between their [M+K]^+^ ions; however, sucrose did not show any peak in the *m*/*z* 180 ion. Glucose (C_6_H_12_O_6_, 180.06) was detected as the [M+K]^+^ ion at *m*/*z* 219 and there were no significant ion at *m*/*z* 180 (Fig. 1c).

To verify the matrix effect in ginkgo stem, the standard chemicals were dissolved in the aqueous extract of ginkgo stem and measured using cryo-TOF-SIMS. The characteristic secondary ions of target chemicals demonstrated using KClaq were enhanced in the spectra of ginkgo extracts (Supplementary Fig. 4 and Supplementary Fig. 5). This result means that the target chemicals produce the same characteristic secondary ions in both matrix solutions of KClaq and ginkgo extract. From these points, it should be possible to obtain individual chemical mappings using the *m*/*z* 180 ion for coniferin, the *m*/*z* 381 ion for sucrose (disaccharide), and the *m*/*z* 219 ion for glucose and fructose (monosaccharide).

### Radial distribution of coniferin in the transverse surface

**Figure 2:**
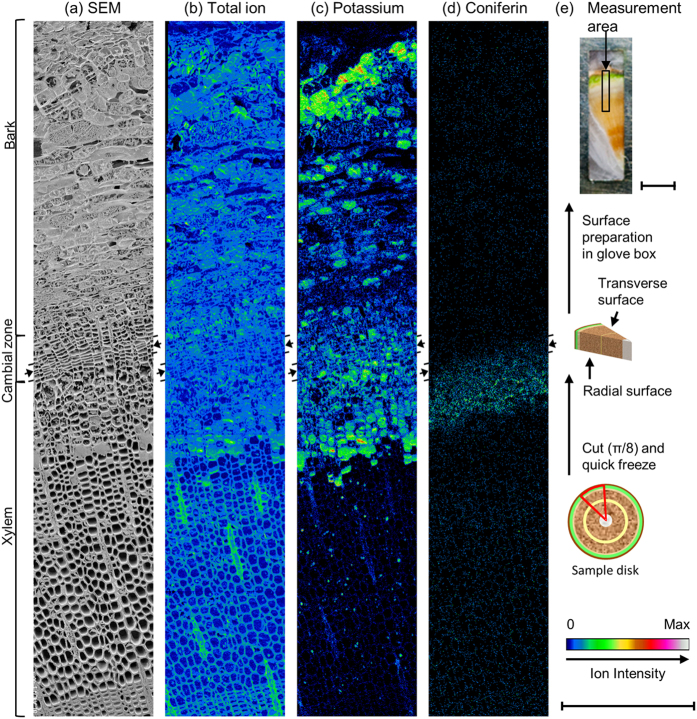
Transverse surface images of freeze-fixed ginkgo stem by cryo-TOF-SIMS/SEM. (**a**) Cryo-SEM image taken after cryo-TOF-SIMS measurement and appropriate freeze-etching. Cryo-TOF-SIMS positive ion images of (**b**) total ion, (**c**) K^+^ at *m*/*z* 39, and (**d**) coniferin at *m*/*z* 180. (**e**) Schematic illustration of sample preparation and the resultant optical microscopic image of transverse surface of freeze-fixed ginkgo stem on a cryo-TOF-SIMS sample holder showing the measurement area (ca. 2.3 × 0.4 mm). Scale bars are 500 μm for (**a–d**) and 2 mm for (**e**). Arrows at both sides of images suggest the line of the cambial zone. Cryo-SEM images before and after freeze-etching are displayed in Supplementary Fig. 6. Cryo-TOF-SIMS images of mono-/di- saccharides are shown in Supplementary Fig. 7.

Figure 2 displays the results of cryo-TOF-SIMS/SEM analysis of the freeze-fixed ginkgo stem. After cryo-TOF-SIMS measurements, the same region of the sample surface was observed using cryo-SEM. Just after cryo-TOF-SIMS measurements, the sample surface maintained its frozen-hydrated state (Supplementary Fig. 6). Therefore, cryo-SEM observations were conducted after appropriate freeze-etching to enhance the contrast of cryo-SEM images. The measured area contains bark, cambial zone, and xylem (Fig. 2a,e).

Potassium (Fig. 2c) was detected in the region from bark to outer xylem and only in the ray cells in a mature xylem region. This observed distribution of potassium is largely similar to that in living tissues. Coniferin was found only in the differentiating xylem region next to the cambial zone (Fig. 2d). Distribution of secondary ions of mono-/di- saccharides partially overlapped with but was not identical to that of coniferin (Supplementary Fig. 7).

**Figure 3:**
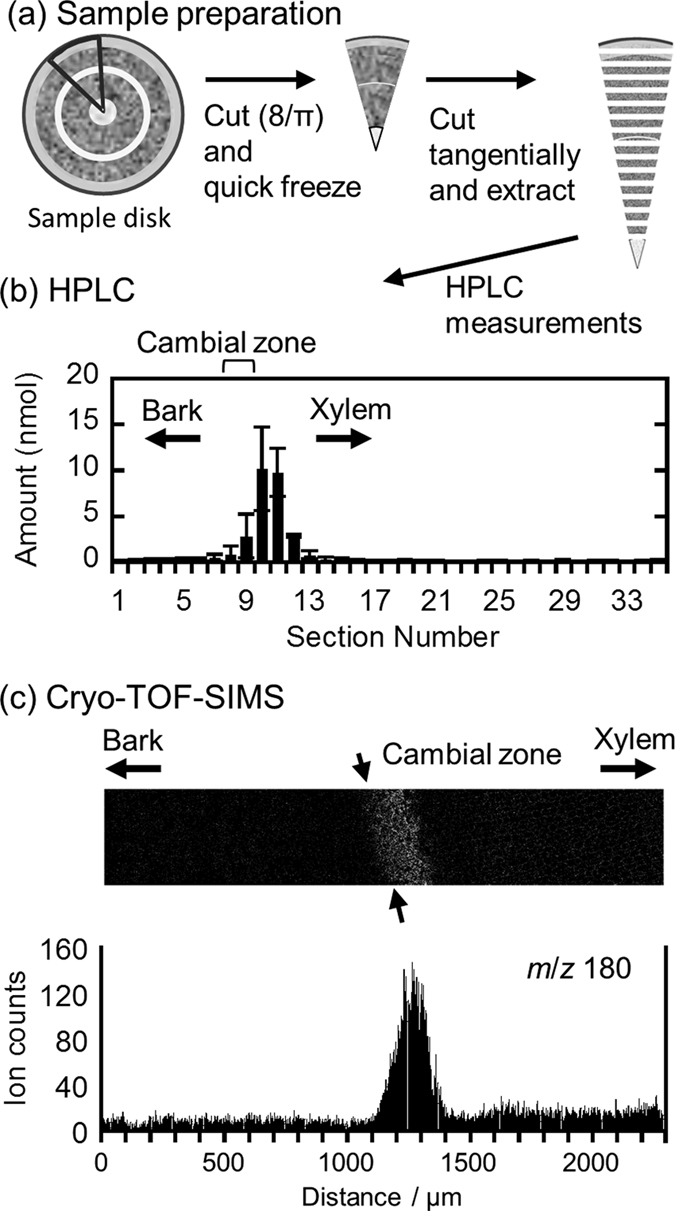
The radial distribution of coniferin evaluated by (**b**) HPLC and (**c**) cryo-TOF-SIMS. Preparation procedure of tangential sections for HPLC measurements is shown in (**a**). In (**b**), serial tangential sections of 100-μm thickness were used and the position of cambial zone corresponding to the section numbers 9 and 10 was determined by the dry weight of the sections as shown in Supplementary Fig. 9. The means and standard deviations for each section in (**b**) were obtained from three sets of measurements using the different sample blocks cut from the same disk. In (**c**), the *m*/*z* 180 ion count from bark to xylem was used and arrows at the distance 1100 μm indicate the position of the cambial zone.

To confirm the actual amount and rough radial distribution of coniferin, a freeze-fixed ginkgo block (circular sector of π/8) was cut into serial tangential sections, and the sections were extracted using hot water. Coniferin in each section was quantified using high performance liquid chromatography (HPLC), and its distribution is summarized in Fig. 3a (see Supplementary Fig. 8 and Supplementary Fig. 9 for the quantification results for sucrose, glucose, and fructose by ion chromatography).

Coniferin showed maximum yield at the xylem next to the cambial zone (Fig. 3a). The radial distribution of the *m*/*z* 180 ion count (Fig. 3b) was consistent with the results of the HPLC analysis. Therefore, we believe that the distribution of coniferin was successfully visualized by cryo-TOF-SIMS.

### Intracellular distribution of coniferin in the radial surface

**Figure 4:**
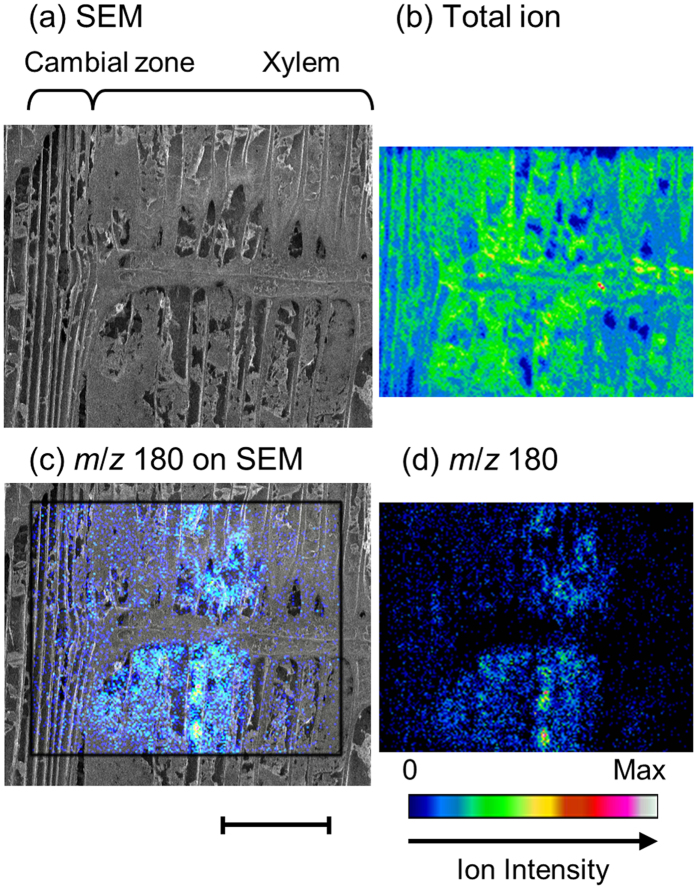
Radial surface images of freeze-fixed ginkgo stem by cryo-TOF-SIMS/SEM. (**a**) Cryo-SEM image after cryo-TOF-SIMS measurement and freeze-etching. Cryo-TOF-SIMS positive ion images of (**b**) total ion and (**d**) *m*/*z* 180 ion. (**c**) The overlay image of cryo-TOF-SIMS *m*/*z* 180 ion on the cryo-SEM image. Scale bar is 100 μm.

Cryo-TOF-SIMS/SEM measurements were also conducted for the radial surface of the freeze-fixed ginkgo stem. Cryo-SEM, cryo-TOF-SIMS total ion and the *m*/*z* 180 ion images are shown in Fig. 4. Furthermore, the *m*/*z* 180 ion mapping was overlaid on the cryo-SEM image to clarify the detailed distribution (Fig. 4c). The overlay image with the cryo-SEM image without freeze-etching is shown in Supplementary Fig. 10. The *m*/*z* 180 ion was detected in most of the inner part of the cell in the differentiating xylem region.

In gymnosperms, the vacuole generally occupies a large part of the volume of the differentiating tracheid cells^7^,^38^,^39^,^40^. Some previous studies suggest the possibility of coniferin being stored in the vacuole^1^,^7^,^41^. Furthermore, it has been recently reported that coniferin tends to be taken into a tonoplast vesicle rather than a plasma membrane vesicle^42^,^43^. Taking into consideration these points, the cryo-TOF-SIMS/SEM image suggests the presence of coniferin storage in the vacuoles of differentiating tracheid cells.

The *m*/*z* 180 ion was detected at lower levels in ray cells. This result does not eliminate the possibility of coniferin transfer via ray cells, because cryo-TOF-SIMS can visualize the concentration of the chemical only at the moment of freezing. Here we can simply conclude that coniferin was not stored in ray cells.

### Semi-quantitative cellular distribution of coniferin

Coniferin was detected in a wide area of the radial section of the tracheid cells in the outer xylem region. To compare the quantity of coniferin in various parts of the differentiating xylem, we evaluated the relative cellular content of coniferin using the cryo-TOF-SIMS results obtained for the transverse surface. Regarding relative quantification in SIMS analyses, the possibility of the presence of inorganic and organic cations in frozen-hydrated samples has been reported^44^,^45^.

**Figure 5:**
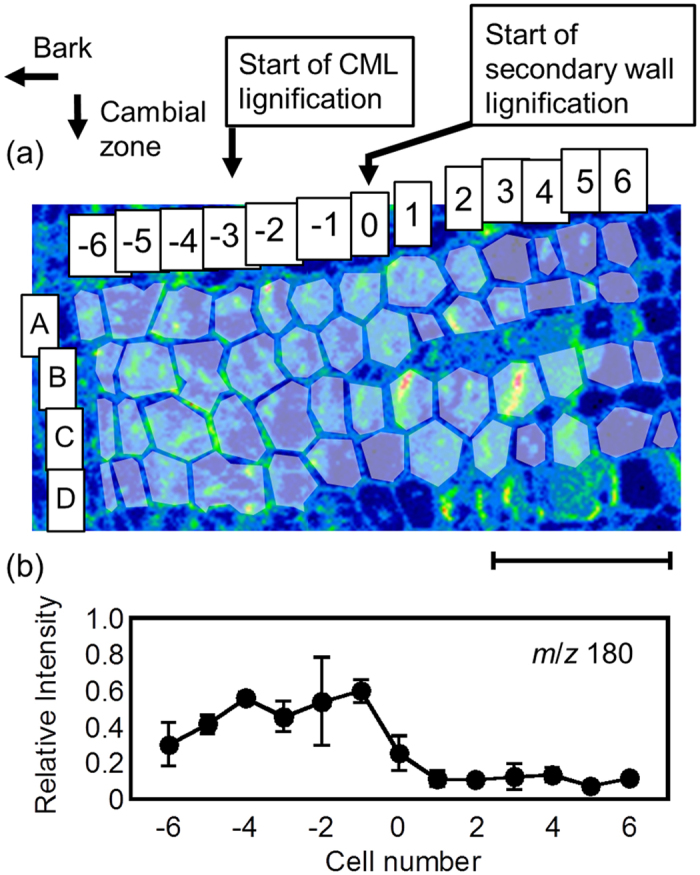
(**a**) The enlarged total ion image of cryo-TOF-SIMS demonstrating the generation of ROIs (n = 4 using the lines A, B, C, and D). Resultant relative ion intensities are summarized in (**b**). Scale bar is 100 μm.

Relative intensity of the *m*/*z* 180 ion was evaluated for each cell using region-of-interest (ROI) analysis of cryo-TOF-SIMS data (Fig. 5a) with eq (1).


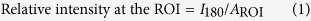


where *I*_180_ is the *m*/*z* 180 ion count in the ROI and *A*_ROI_ is the pixel area of the ROI.

The differentiating stages of the cells were determined with reference to microscopic observations using visible, polarized, and UV light (Supplementary Fig. 11). Secondary wall lignification had started at the cells in column 0 (Fig. 5a). The cells in column −3 correspond to the commencement of compound middle lamella (CML) lignification and the cells in column −6 represent the end of the cambial zone. ROIs were prepared for each cell in columns −6 to 6 and lines A to D. The average and standard deviation of the relative intensity of the *m*/*z* 180 ion were evaluated using these defined ROIs. Resultant relative intensity of the *m*/*z* 180 ion (Fig. 5b) suggests the transition of coniferin concentration in the differentiating tracheid cells.

The relative intensity of the *m*/*z* 180 ion increased gradually from column −6. After the dip in intensity at the CML lignification stage (column −3), the relative intensity of the *m*/*z* 180 ion increased again. Finally, the *m*/*z* 180 ion drastically decreased at the start of secondary wall lignification (column 0). In the inner columns, after the diminution (column 1–6), the *m*/*z* 180 ion was detected only at very low levels. This correlation between coniferin storage and the lignification stage will be discussed further with respect to coniferin assimilation timing.

### Coniferin distribution and lignification stages

Administered ^13^C-labelled L-phenylalanine is effectively converted to monolignol glucoside in differentiating xylem of ginkgo^8^. It was also demonstrated that the administered ^13^C or ^14^C-labelled monolignol glucosides is efficiently incorporated into lignin if they are fed to differentiating xylem of various plants^1^,^4^,^6^,^8^,^9^,^10^,^11^,^12^,^13^,^14^,^15^,^16^,^17^. From these results, it can be deduced that monolignol glucosides are real intermediate compounds in lignin biosynthesis and are naturally assimilated to lignin on condition that they exist in the lignifying cell.

**Figure 6:**
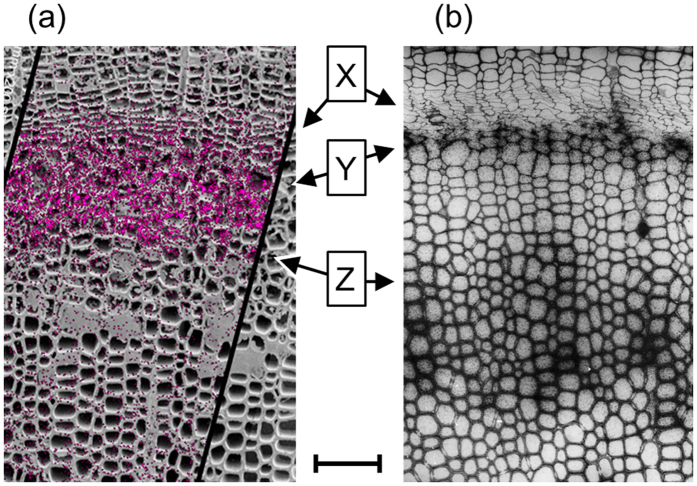
Comparative visualization of (**a**) the overlay image of cryo-TOF-SIMS *m*/*z* 180 ion (red) on the cryo-SEM illustrating endogenous coniferin distribution in the freeze-fixed ginkgo stem and (**b**) ^14^C microautoradiography showing ^14^C-lignin introduced by ^14^C-coniferin administration to ginkgo (rearranged from the previous research by Fukushima and Terashima^11^). Arrows show the positions of (X) cambial zone, (Y) start of CML lignification, and (Z) start of secondary cell wall lignification. Scale bar is 100 μm.

In this study, the cellular distribution of endogenous coniferin was visualized semi-quantitatively for the first time. The results agree with the previous results of two-step assimilation of administered coniferin to lignin (Fig. 6). The amount of endogenous coniferin increased within the cell wall thickening period even in CML lignification (Y of Fig. 6a), and diminished at the secondary wall lignification stage (Z of Fig. 6a). On the other hand, administered coniferin was assimilated to lignin at the commencement of CML lignification (Y of Fig. 6b) and secondary wall lignification (Z of Fig. 6b). These results suggest a common description of the coniferin behaviour in the differentiating xylem.

Additionally, it should be noted that secondary wall lignification might occur at several lines of cells simultaneously (inner region next to Z of Fig. 6). The cells at the corresponding region in the cryo-SEM image had some contents suggesting their living state (inner region next to Z of Fig. 6a). Lower levels of coniferin were detected at the region probably because of the low concentration of coniferin in the cells (Fig. 5). Finally, the tracheid cells completed lignification and died. The living period of tracheid cells was also confirmed by the potassium distribution (Fig. 2c) suggesting living cells.

## Conclusion

In summary, cryo-TOF-SIMS/SEM analysis visualized water-soluble chemicals *in planta* with subcellular resolution. Semi-quantitative cellular distribution of coniferin showed good agreement with the assimilation timing of coniferin to lignin in differentiating xylem previously visualized by ^14^C-labelled coniferin administration. These results lead us to the conclusion that coniferin is stored in the tracheid cells of differentiating xylem and is utilised as a lignin precursor. The intercellular and intracellular coniferin transport and the subsequent use of the residual glucose unit of coniferin is the next important topic for investigation.

## Methods

### Plant Materials

The sample disk (thickness 10 mm) was obtained from a two-year-old flesh shoot of ginkgo and cut into small blocks (circular sector of radius 5 mm and central angle π/8) containing bark, cambial zone, and xylem on 19th June 2014 in Nagoya, Japan. The blocks were quick-frozen with liquid Freon^®^ 22 (DuPont) at −160 °C and stored at −80 °C.

### Reagents

Coniferin and stable isotope-labelled coniferin (^13^C at α position of the aglycon) were synthesized by the same method as described by Terashima *et al.*^46^

### Chromatography measurements

A frozen block was cut to serial tangential sections of 100 μm thick from bark to xylem. Each section was extracted using 1 mL water for 30 min at 95 °C and 2.5 h at r.t. The extracts obtained were analysed by HPLC to quantify coniferin. HPLC measurements were performed using a Shimadzu SPD-10A apparatus. The measuring conditions were as follows: Column: TSK-gel ODS-100S (4.6 mmID × 250 mm, Tosoh corp., Japan); flow rate: 1.0 mL min^−1^; temperature: 40 °C; eluent: H_2_O (solvent A) and methanol:acetonitrile = 6:1 [v/v] (solvent B) with a gradient of B 10% 10 min, B 10–20% 10 min, B 20–60% 10 min, B 60–10% 5 min, B 10% 10 min.

### Cryo-TOF-SIMS/SEM analyses

The detail of the manufactured cryo-TOF-SIMS/SEM system was as described previously by Kuroda *et al.*^35^ and Masumi *et al.*^47^. The frozen sample block was fixed in a Cu sample holder by ice-embedding. After cutting to form a clean and flat surface in the glove box under dry N_2_ atmosphere (<−10 °C), the block was transferred to cryo-TOF-SIMS by a cryo-vacuum shuttle. Positive ion images were obtained by cryo-TOF-SIMS (TRIFT III spectrometer, ULVAC-PHI Inc.). Measurement conditions were as follows: primary ion, 22 keV Au_1_^+^ at a current 5 nA; raster size (pixel resolution), 400 μm × 400 μm (1.56 μm for images) or 200 μm × 200 μm (0.78 μm for spectra); pulse width, 13.0 ns (non-bunched for image) or 1.8 ns (bunched for spectrum); mass range, *m*/*z* 0.5–1850; spot size, 1.0 μm in image mode; temperature, −120 to −130 °C; a low-energy pulsed electron ion gun (30.0 eV) was used for surface charge compensation. All images accumulated 3,000,000 secondary ion counts in non-bunched mode and primary ion doses for the sample surface were 1.4–1.6E + 9/cm^2^ for the region containing bark to differentiating xylem and 2.5–2.6E + 9/cm^2^ for the region containing only mature xylem. Furthermore, bunched mode measurements were conducted for the same ginkgo sample to check the peak unimodality and *m*/*z* calibration. As a result, the *m*/*z* 180, 219, and 381 ion peaks were unimodal in both measurement modes. Only the *m*/*z* 39 ion peak was bimodal in both measurement modes. The bimodal *m*/*z* 39 ion peak consisted of dominant K^+^ (*m*/*z* 38.96) and minor C_3_H_3_ (*m*/*z* 39.02) ions and it was possible to separate K^+^ ion in non-bunched mode spectra. Consequently, we concluded that we could visualize target ions in non-bunched mode in this study.

Subsequently to the cryo-TOF-SIMS measurements of freeze-fixed ginkgo stem, the same region was observed by cryo-SEM. Because the sample surface maintained its frozen-hydrated state after cryo-TOF-SIMS measurements, freeze-etching treatment was applied to enhance the contrast of cryo-SEM images (Supplementary Fig. 6 and Supplementary Fig. 10). The observation conditions were as follows: acceleration voltage, 1.5 kV; temperature, −120 °C for observation and −90 °C for freeze-etching; working distance 10 mm. Aqueous solutions of standard chemicals were frozen and analysed using cryo-TOF-SIMS employing the same procedure in bunched mode.

All TOF-SIMS data were obtained as ‘RAW’ data files recording a full mass spectrum at every 256 × 256 pixel point. Obtained images are connected by using WinCadence 5.1.2.8 (ULVAC-PHI Inc.) and MatLab R2014a (The MathWorks, Inc.) with PLS Toolbox 7.5.2 (Eigenvector Research, Inc.), and the colour scale was changed by ImageJ software (The National Institutes of Health, USA, http://rsb.info.nih.gov/ij/). The region of interest (ROI) function can produce mass spectral datum from areas defined by using drawing tools in an image acquired as raw data by using WinCadence 5.1.2.8. The ROI analysis was done using the RAW file containing cambial zone and differentiating xylem region obtained by 1.6E + 9/cm^2^ primary ion dose. SEM images and overlay images of the selected ion on SEM image were prepared by using Photoshop CS5 Extended (Adobe Systems Incorporated).

## Additional Information

**How to cite this article**: Aoki, D. *et al.* Distribution of coniferin in freeze-fixed stem of *Ginkgo biloba L.* by cryo-TOF-SIMS/SEM. *Sci. Rep.* **6**, 31525; doi: 10.1038/srep31525 (2016).

## Supplementary Material

Supplementary Information
