# Supplementary material for: Distribution of coniferin in freeze-fixed stem of Ginkgo biloba L. by cryo-TOF-SIMS/SEM
Source: Sci Rep. 2016 Aug 11;6:31525. doi: 10.1038/srep31525 (PMC4980676; doi:10.1038/srep31525)
Supplement: Supplementary Information [file srep31525-s1.pdf]

Supplementary Information

Distribution of coniferin in freeze-fixed stem of *Ginkgo biloba L.* by cryo-TOF-SIMS/SEM

Dan Aoki[1*], Yuto Hanaya[1], Takuya Akita[1], Yasuyuki Matsushita[1], Masato Yoshida[1], Katsushi Kuroda[2], Sachie Yagami[1], Ruka Takama[1], and Kazuhiko Fukushima[1]

[1] Graduate School of Bioagricultural Sciences, Nagoya University, Furo-cho, Chikusa-ku, Nagoya, Aichi 464-8601, Japan

[2] Department of Wood Properties and Processing, Forestry and Forest Products Research Institute, 1 Matsunosato, Tsukuba, Ibaraki 305-8687, Japan

* Corresponding author

E-mail address: daoki@agr.nagoya-u.ac.jp

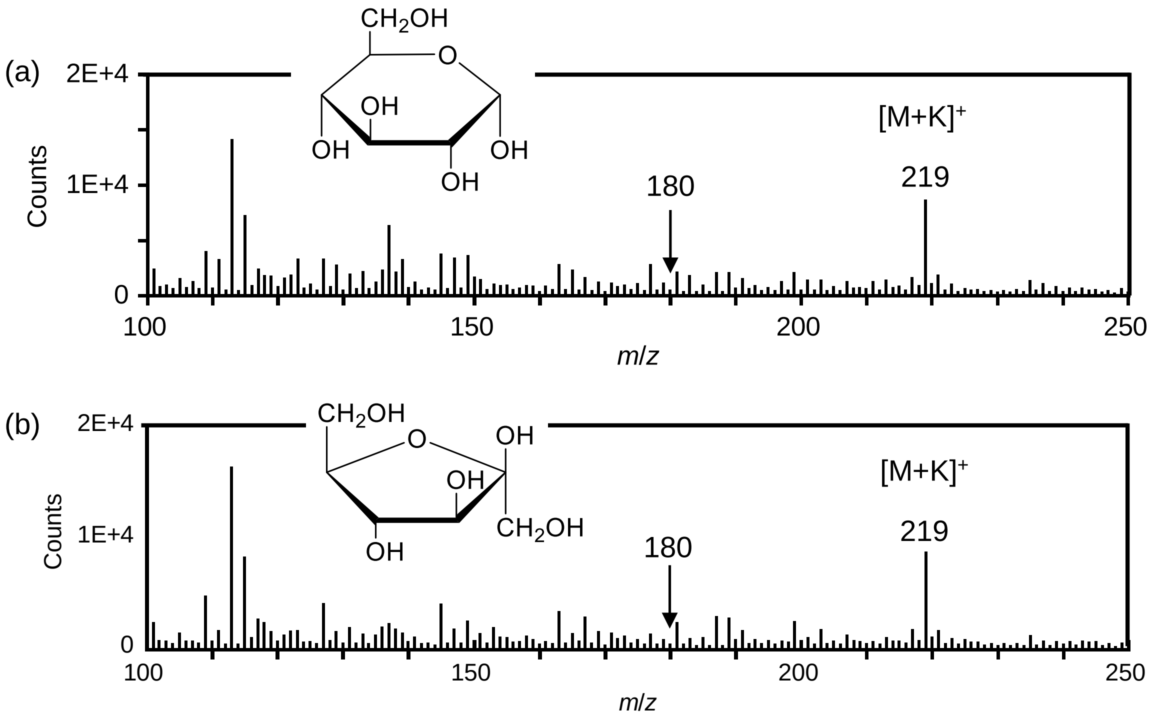

Supplementary Fig. 1 Cryo-TOF-SIMS spectra of (a) D-glucose and (b) D-fructose.

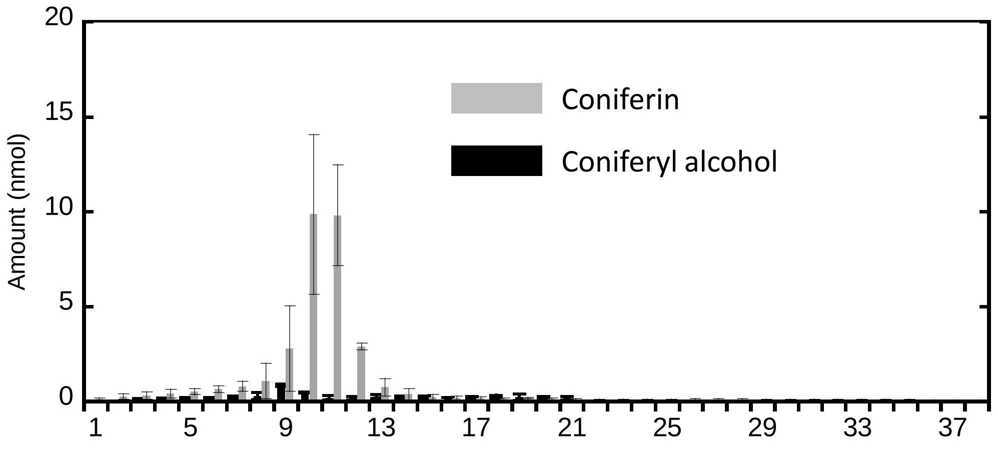

Supplementary Fig. 2 Result of HPLC quantification of coniferin and coniferyl alcohol

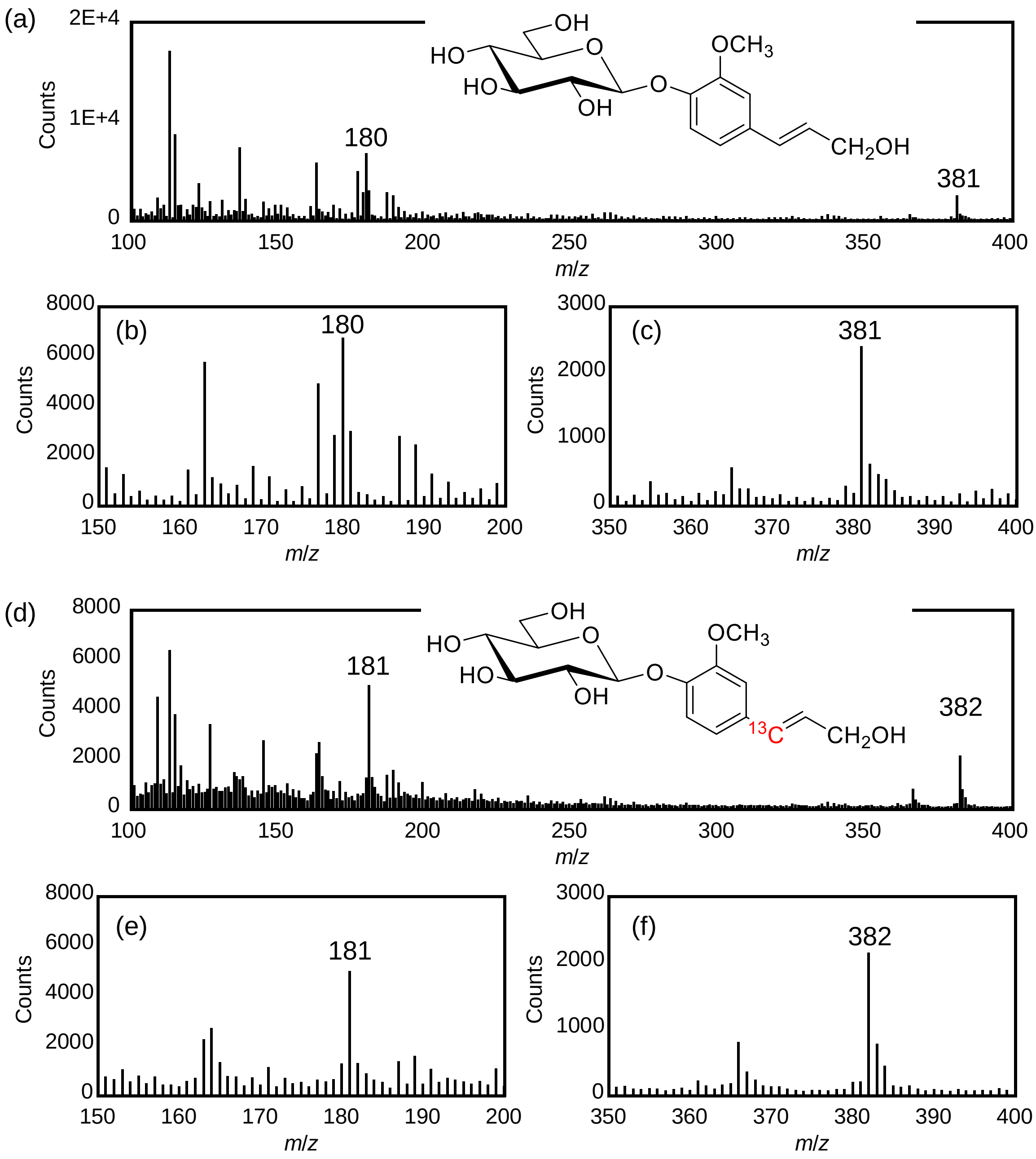

Supplementary Fig. 3 Cryo-TOF-SIMS spectra of (a,b,c) coniferin and (d,e,f) ^{13}C-labelled coniferin. Expanded spectra were shown for the *m*/*z* 150–200 and *m*/*z* 350–400 regions.

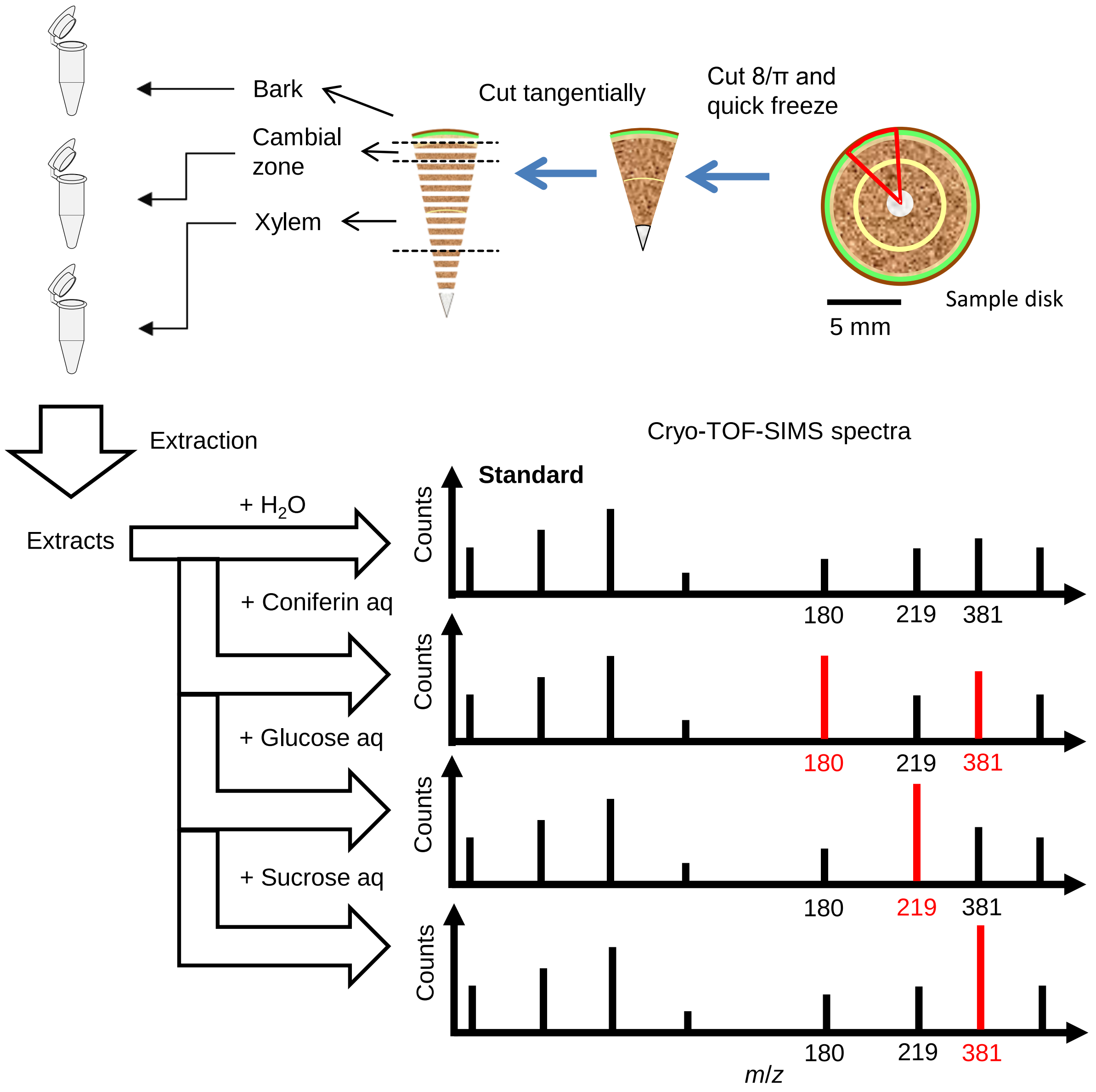

Supplementary Fig. 4 Schematic illustration of the experimental examination of matrix effect on the ionization and the fragmentation behaviour of coniferin, glucose, and sucrose in different extracted solutions obtained from bark, cambial zone, and xylem regions of ginkgo stem.

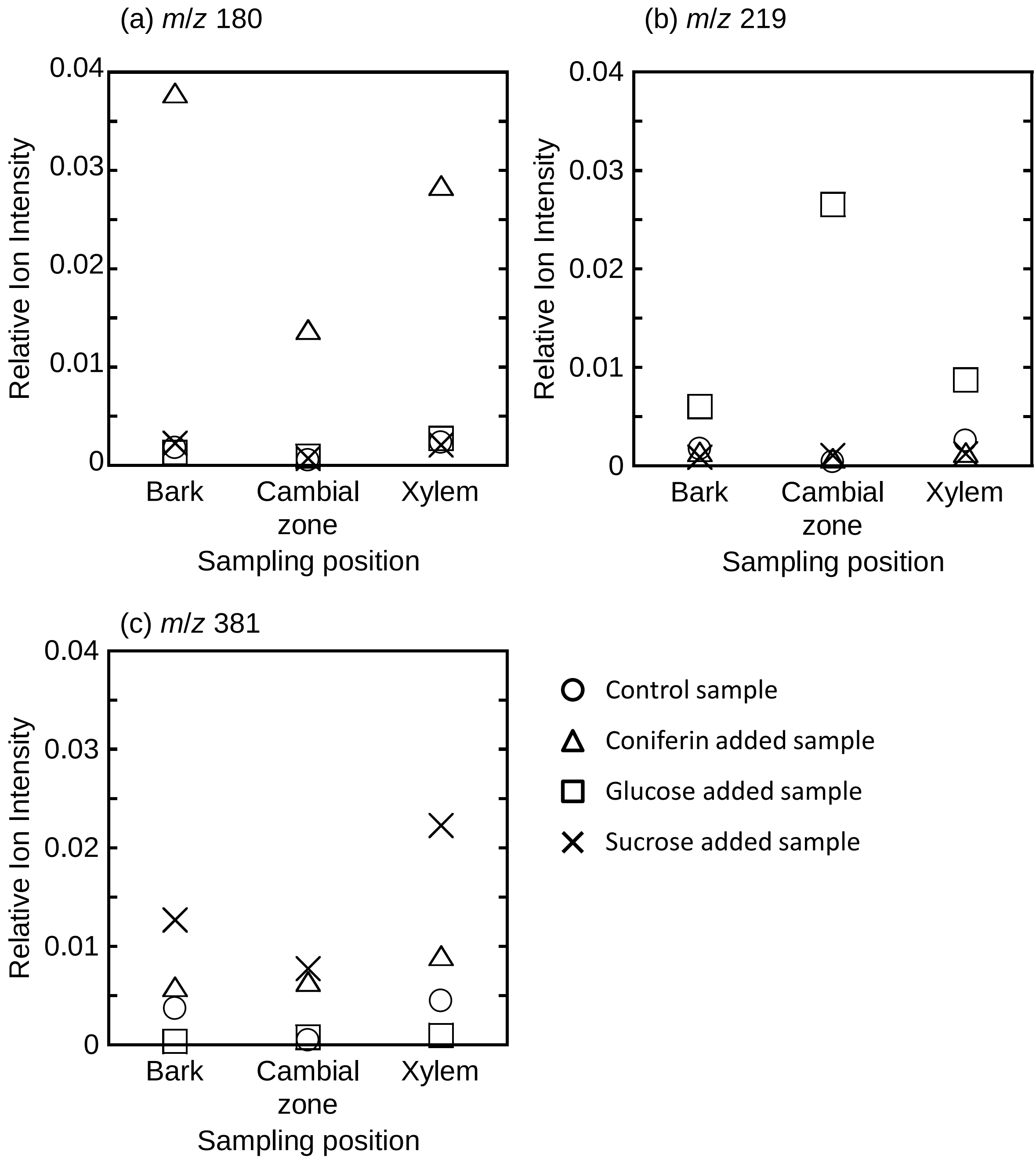

Supplementary Fig. 5 Relative ion intensity of *m*/*z* 180, 219, and 381 ions using extract samples from bark, cambial zone, and xylem regions. Coniferin, glucose, or sucrose was added to each extract and the relative ion intensities were compared with those of control sample.
(a) *m*/*z* 180 ion increased only by coniferin addition.
(b) *m*/*z* 219 ion increased only by glucose addition.
(c) *m*/*z* 381 ion increased by coniferin or sucrose addition.
The ion yield per mol of sucrose was higher than that of coniferin for *m*/*z* 381 ion. Furthermore, the actual amount of sucrose was much higher than that of coniferin in ginkgo (Supplementary Fig. 8). From these points, the distribution of *m*/*z* 381 ion should be derived mainly from sucrose.

(a)

(b)

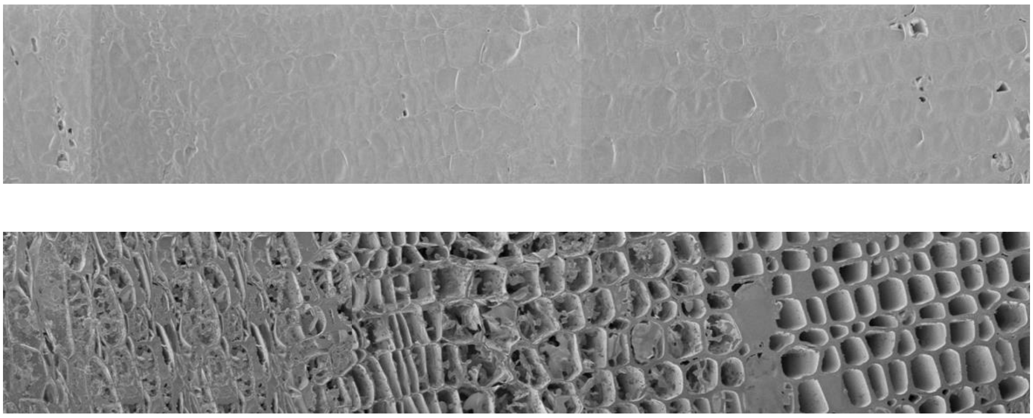

Supplementary Fig. 6 Cryo-SEM images of transverse surface of freeze-fixed ginkgo stem (a) just after cryo-TOF-SIMS measurements and (b) after freeze etching. Cryo-SEM image shows that there was almost no surface sublimation within the cryo-TOF-SIMS measurements at -120 °C.

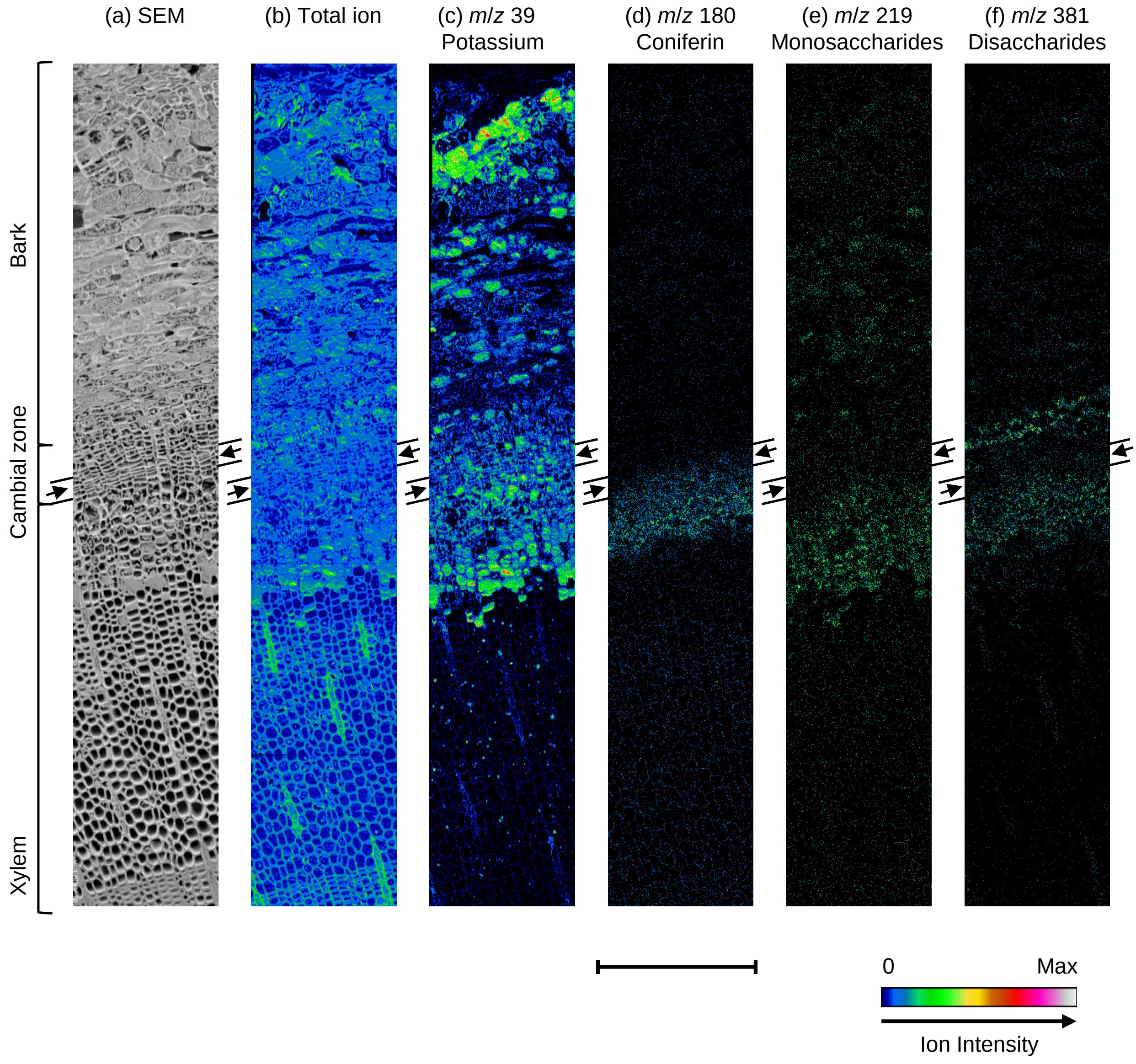

Supplementary Fig. 7 Transverse surface images of freeze-fixed ginkgo stem by cryo-TOF-SIMS/SEM. (a) Cryo-SEM image taken after cryo-TOF-SIMS measurement and appropriate freeze-etching. Cryo-TOF-SIMS positive ion images of (b) total ion, (c) K^+ at *m*/*z* 39, (d) coniferin at *m*/*z* 180, (e) monosaccharides (glucose and fructose) at *m*/*z* 219, and (f) disaccharides (sucrose) at *m*/*z* 381. Scale bar is 500 µm. Arrows at both sides of images suggest the line of the cambial zone.

As have been mentioned (Supplementary Fig. 5), *m*/*z* 381 ion should be derived mainly from sucrose. In fact, the distribution of *m*/*z* 180 ion (coniferin) and *m*/*z* 381 ion (disaccharides) were different.

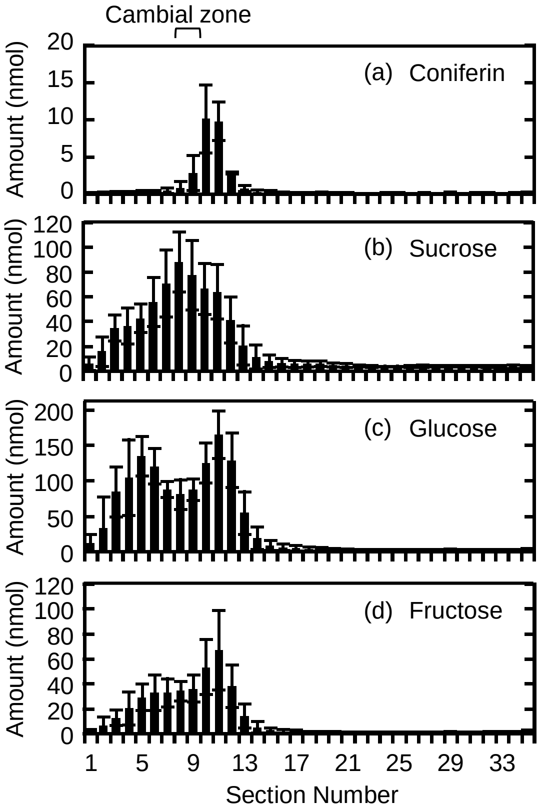

Supplementary Fig. 8 The radial distribution of (a) coniferin evaluated by HPLC and (b) sucrose, (c) glucose, and (d) fructose evaluated by ion chromatography using serial tangential sections of 100-μm thickness. The means and standard deviations for each section were obtained from three sets of measurements using the different sample blocks cut from the same disk. The position of cambial zone corresponding to the section numbers 9 and 10 was determined by the dry weight of the sections as shown in Supplementary Fig. 9.

Ion chromatography measurements were conducted using a DIONEX ICS-3000 apparatus. The measuring conditions were as follows: column, CarboPac PA-1 (2.0 mmID × 250 mm, Dionex corp.); flow rate, 0.3 mL min−1; temperature, 30 °C; eluent, H2O (solvent A), 100 mM NaOHaq (solvent B), and aqueous solution containing 100 mM NaOH and 1.0 M CH3COONa (solvent C) with a gradient of B 50% C 0 % 50 min, C 100 % 10 min, B 100 % 10 min, B 50 % C 0% 15 min.

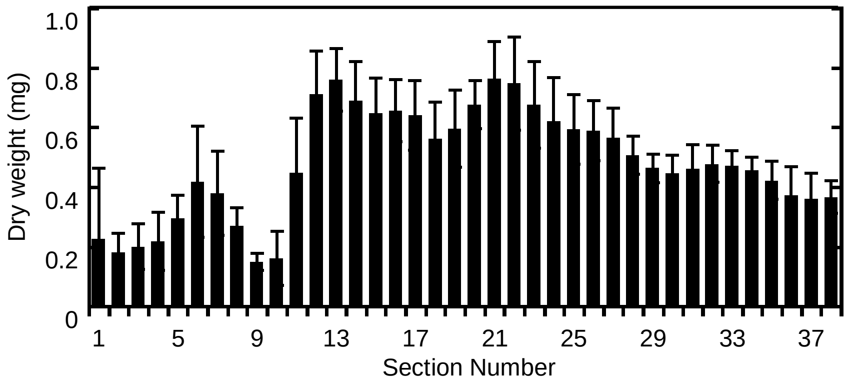

Supplementary Fig. 9 Dry weights of extracted serial tangential sections. Sections 9 and 10 were determined as the sections containing cambial zone.

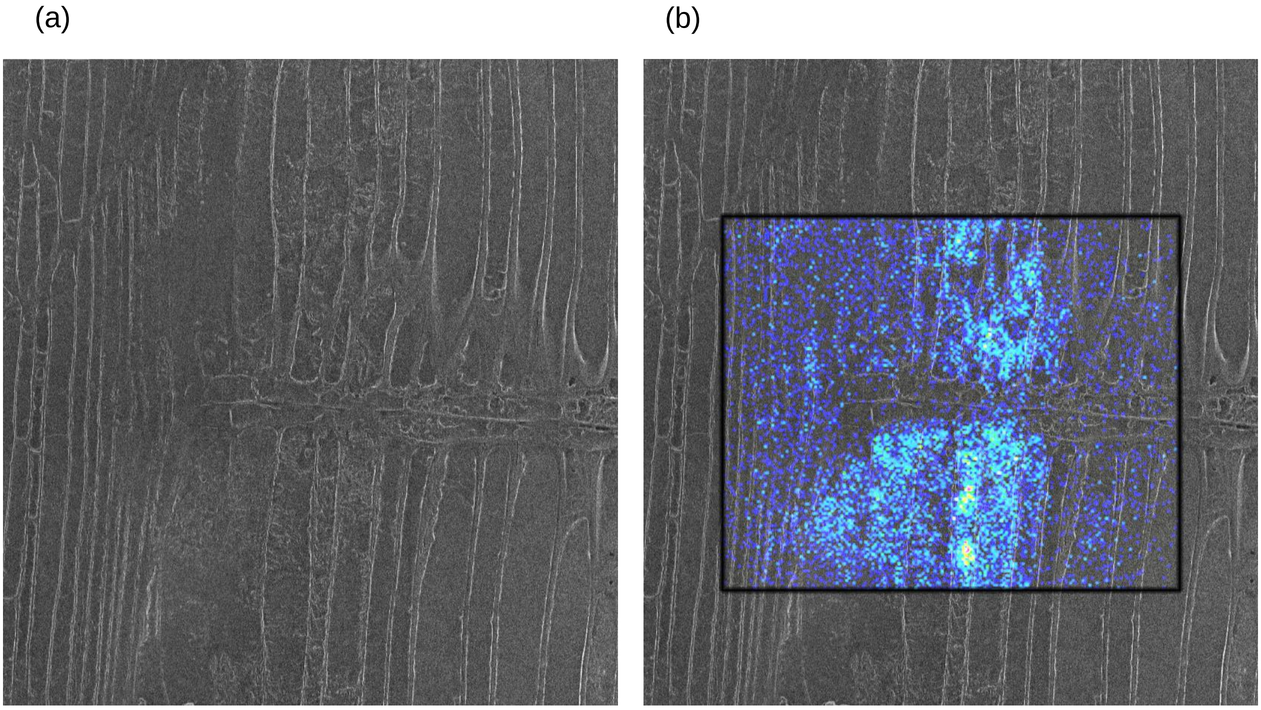

Supplementary Fig. 10 (a) Cryo-SEM image just after cryo-TOF-SIMS measurement and (b) the overlay image of cryo-TOF-SIMS *m*/*z* 180 ion on the cryo-SEM image.

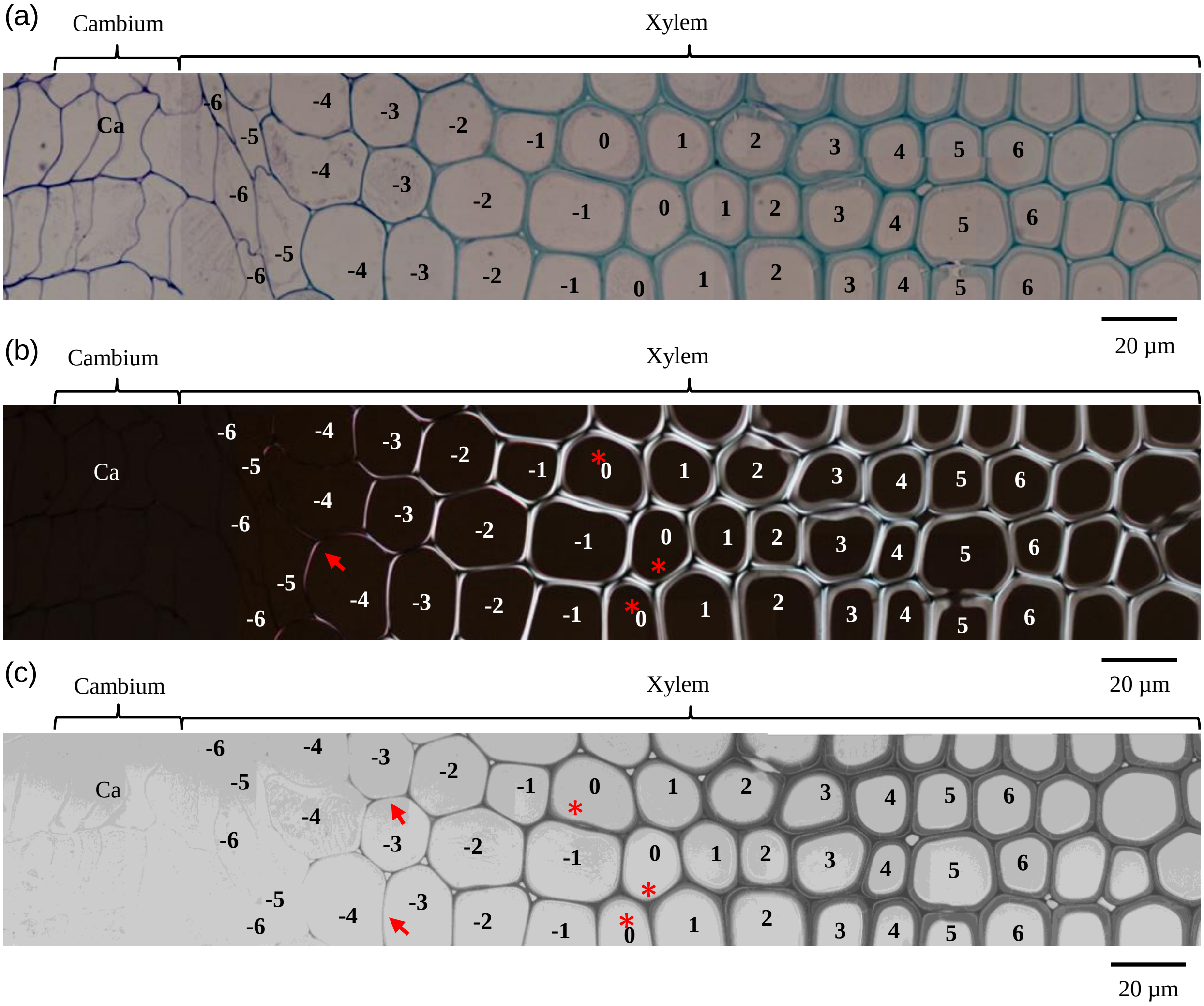

Supplementary Fig. 11 Images of transverse section of resin-embedded ginkgo stem observed by (a) visible, (b) polarized, and (c) UV lights. An arrow in (b) shows the end of S1 layer birefringence, arrows in (c) suggest the start of CML lignification, and asterisks suggest the start of secondary wall lignification.
